# The role of AMP-activated protein kinase in GVHD-causing T cells

**DOI:** 10.1097/IN9.0000000000000009

**Published:** 2022-10-07

**Authors:** Archana Ramgopal, Lee-Kai Sun, Craig A. Byersdorfer

**Affiliations:** 1 Division of Blood and Marrow Transplant and Cellular Therapies, Department of Pediatrics, University of Pittsburgh School of Medicine, Pittsburgh, PA, USA

**Keywords:** T cell metabolism, oxidative metabolism, AMP-activated protein kinase, AMPK, blood and marrow transplantation, GVHD

## Abstract

Allogeneic stem cell transplantation is a curative therapy for multiple hematologic disorders. However, this life-saving procedure is often complicated by acute graft-versus-host disease (GVHD), where donor T cells attack tissues in the recipient’s skin, liver, and gastrointestinal tract. Previous research has demonstrated that GVHD-causing T cells undergo significant metabolic reprogramming during disease pathogenesis, with an increased reliance on oxidative metabolism. This dependence makes metabolic modulation a potential approach to treat and/or prevent GVHD. Here, we provide an overview on the metabolic changes adopted by allogeneic T cells during disease initiation, highlighting the role played by AMP-activated protein kinase (AMPK) and identifying ways in which these insights might be leveraged to therapeutic advantage clinically.

## 1. Introduction

Allogeneic hematopoietic stem cell transplantation (alloHSCT) is a cellular therapy that provides long-term cures to patients with a myriad of hematologic disorders including relapsed and refractory leukemia ^[1]^. However, the therapeutic use of alloHSCT remains limited by acute graft-versus-host disease (GVHD), where activated donor T cells attack and destroy host tissues in the skin, gastrointestinal tract, and liver ^[2]^. GVHD occurs in 30% to 50% of allogeneic transplants and represents a leading cause of non-relapse morbidity and mortality ^[3]^. The first-line therapy for acute GVHD remains corticosteroids, however sustainable remissions occur in fewer than 50% of patients ^[4]^, and steroid treatment can result in multiple side effects including hyperglycemia, hypertension, and increased risk of infection ^[5]^. In addition, many current GVHD treatments target T cells in an indiscriminate fashion, undermining the effectiveness of graft-versus-leukemia (GVL) responses and increasing rates of relapse ^[6,7]^. Thus, new therapies to mitigate GVHD, while still preserving homeostatic immune reconstitution and robust anti-leukemic activity, are urgently needed.

Metabolic manipulation of donor T cells offers an innovative approach to modulate GVHD severity. Early studies demonstrated that alloreactive T cells undergo metabolic reprogramming early post-transplant including adoption of oxidative metabolism ^[8,9]^ and increased rates of fatty acid oxidation (FAO) ^[10]^. AMP-activated protein kinase (AMPK) is a cellular energy sensor that promotes oxidative metabolism downstream of T cell receptor engagement and blocks energy-consuming in cells when nutrients are limited ^[11]^. In murine transplant studies, AMPK has been implicated as a driver of GVHD, and deletion of AMPK in donor T cells protects against the development of severe disease ^[9,12]^. This brief review will describe what is understood about oxidative metabolism in T cells, highlight AMPK as a prospective target in mitigating post-transplant GVHD severity, and identify potential strategies to modulate AMPK clinically in patients.

## 2. Oxidative metabolism in T cells

Oxidative phosphorylation (OXPHOS) generates ATP in the mitochondria via creation of a proton gradient and subsequent transport of hydrogen ions through the F_1_F_0_-ATPase, the terminal step of the electron transport chain (ETC). Multiple substrates feed into the tricarboxylic acid (TCA) cycle, where serial metabolic processing of acetyl-CoA molecules generates reducing substances NADH and FADH_2_^[13]^, which in turn donate electrons to the ETC to create the initial proton gradient. Classically, use of oxidative metabolism has been thought to depend on the differentiation and functional state of the T cell. In this construct, naive T cells rely predominantly on OXPHOS prior to antigenic stimulation ^[14]^, acutely activated T cell upregulate glycolysis, while both memory (Tmem) and regulatory T cells (Treg) exhibit higher levels of OXPHOS to execute their respective functions ^[15]^.

Depending on the environmental context, oxidative metabolism in T cells may have either therapeutic or pathological effects. Adoption of autophagy and FAO by Tmem promotes their rapid response to antigenic re-challenge ^[16,17]^. In Treg cells, expression of Foxp3 is linked to mitochondrial respiration and FAO, changes which facilitate their suppressive function ^[18,19]^. In chimeric antigen receptor (CAR) T cells, a cellular immunotherapy utilized in the treatment of hematologic malignancies, the degree of OXPHOS correlates with the therapeutic efficacy of the T cell ^[20]^. CARs designed with a 4-1BB signaling domain exhibit increased mitochondrial biogenesis ^[21]^ and simultaneously persist longer in vivo ^[22,23]^. Furthermore, specific changes to the manufacturing conditions can skew CAR T cells toward higher oxidative metabolism in vitro ^[24]^; however, whether these phenotypes persist in vivo following CAR T cell infusion remains an open question ^[24]^. T cell malignancies also favor an increase in oxidative metabolism. In T cell acute lymphoblastic leukemia (T-ALL), an aggressive malignancy with a poor prognosis and frequent rates of relapse, mutations in Notch signaling activate the AMPK pathway, which then increases OXPHOS and fuels subsequent leukemia growth ^[25,26]^. Transfer of primary T-ALL cells lacking AMPK into irradiated recipients decreased T-ALL in secondary lymphoid organs and improved recipient survival compared to transfer of WT T-ALL ^[25]^. Oxidative metabolism also drives disease pathology in some T cell–mediated autoimmune disorders. For example, in systemic lupus erythematosus (SLE), an autoimmune condition where autoreactive CD4^+^ T cells promote autoantibody formation by B cells, pathogenic T cells increase glucose oxidation and subsequent glucose processing through the TCA cycle ^[27]^. Inhibition of both glucose metabolism and the ETC in a murine lupus model reduces disease severity and decreased immune complex deposition in the glomerulus ^[28]^.

## 3. Oxidative metabolism in GVHD

In GVHD, alloreactive donor T cells mount pathologic immune responses against healthy recipient tissue. In contrast to the conventional metabolic paradigm, where activated T cells predominantly rely on aerobic glycolysis for rapid cell growth and effector function ^[29]^, activated T cells recovered during GVHD promote both OXPHOS and aerobic glycolysis ^[9]^, a scenario mimicked by a subset of CD8 T cells responding to antigen-bearing *Listeria monocytogenes* in vivo ^[30]^. It is likely that upregulation of OXPHOS in alloreactive T cells is a result of higher energy demands brought about by chronic antigen stimulation in the context of an inflammatory milieu ^[31]^. Gatza et al ^[9]^ showed in murine models that syngeneic bone marrow (BM) cells primarily utilized aerobic glycolysis during cell division, with 2- and 3-fold increases in GLUT1 expression and lactate production respectively, while oxygen consumption remained at basal levels. In contrast, alloreactive T cells demonstrated increases in both GLUT1 and lactate production, but also had a 2-fold increase in oxygen consumption, hyperpolarization of their mitochondrial membrane, and a decrease in antioxidant stores. Oxidative metabolism was necessary in alloreactive T cells as demonstrated by the ability of Bz-423, an inhibitor of the mitochondrial F_1_F_0_-ATPase, to decrease GVHD severity and improve survival in allogeneic recipients compared to vehicle controls.

## 4. Function of AMPK in T cells

The cellular energy sensor AMPK is a heterotrimeric protein kinase complex consisting of a serine/threonine kinase α-subunit, a stabilizing β-subunit, and a regulatory γ-subunit ^[32,33]^. In settings of low cellular energy, binding of ADP and AMP to the regulatory y-subunit promotes phosphorylation of AMPK by upstream kinases ^[11,34,35]^. This activation of AMPK in turn increases oxidative metabolism and upregulates FAO in multiple tissues. In skeletal muscle, AMPK phosphorylation of acetyl-CoA carboxylase (ACC) decreases ACC enzymatic activity and limits malonyl-CoA accumulation. This lack of malonyl-CoA releases the allosteric inhibition of carnitine palmitoyltransferase 1a (CPT1a), the rate-limiting step of FAO, allowing oxidation of fat to proceed ^[33,36]^. Other actions of AMPK include inhibition of the mammalian target of rapamycin (mTOR) signaling pathway and blockade of mRNA translation through phosphorylation of tuberous sclerosis complex 2 (TSC2) and the mTOR subunit, Raptor ^[37–39]^. AMPK also promotes mitophagy and autophagy through phosphorylation of Unc51-like kinase (ULK-1) ^[33]^.

Although T cell development and differentiation depend on specific metabolic pathways, the exact role of AMPK in many facets of T cell biology is still emerging. Early studies demonstrated that AMPK activation is transiently induced upon anti-CD3/CD28 stimulation ^[40,41]^, was not critical for mature T cell homeostasis, and regulates T cell function in contexts of nutrient depletion and metabolic stress ^[42,43]^. Mayer et al ^[42]^ demonstrated that AMPKα1-deficient T cells were more sensitive to inhibition of mitochondrial respiration but exhibited preserved proliferative ability and effector function. Blagih et al ^[43]^ further showed that AMPKα1-deficient T cells maintained proliferative capacity in glucose-rich conditions (25 mM), but decreased their division potential in glucose limited media (3–6 mM). AMPK also shapes T cell differentiation. In CD8^+^ T cells, a lack of AMPK presages poor Tmem formation ^[44,45]^. In a *L. monocytogenes* infection model, AMPKα1-deficient CD8^+^ T cells were comparable to wildtype (WT) T cells during primary infection, but exhibited a decreased response to secondary challenge ^[44]^. T cells lacking TRAF6, an adaptor protein in TNF receptor and TLR signaling pathways, decreased FAO and impaired Tmem formation, a phenotype that recovered following administration of metformin, an AMPK activator ^[45]^.

AMPK has also been implicated in Treg development and function, although there is conflicting evidence regarding its bona fide role in these processes. In murine models, global deletion of AMPK precipitates liver injury, while adoptive transfer of induced, WT Treg prohibited further liver damage ^[46]^. As activated AMPK is known to regulate Foxp3 expression, the controller of Treg stability, specific deletion of AMPKα1 in Treg cells impaired suppressive function ^[46]^. In separate studies, treatment with metformin increased activation of AMPK in Treg cells and elevated percentages of Treg cells in lung-draining lymph nodes during a murine asthma model ^[47]^. However, the degree to which the metformin results were due to AMPK-dependent vs independent functions, for example, metformin’s inhibition of Complex I of the ETC ^[48]^, is unclear. In addition, He et al ^[49]^ reported that LKB1, a direct regulator of AMPK, and not AMPK itself, was responsible for Treg metabolism and survival. In this system, deletion of LKB1 decreased Treg viability, while downstream inhibitors MARKS and SIKS mediated LKB1 function in Treg independent of AMPK ^[49]^. However, these differences may relate to a lack of stressors and the environmental signals normally experienced in vivo, triggers that could skew Treg toward being more reliant on AMPK.

## 5. Deletion of AMPK ameliorates GVHD following murine allogeneic transplantation

Given the role that oxidative metabolism plays in a variety of T cell pathologies, recent studies have begun to examine AMPK and its effects in GVHD-causing T cells. Lepez et al ^[12]^ found that AMPK is not required for early T cell activation and proliferation, but was necessary for sustained T cell expansion. AMPK-deficient T cells proliferated similarly to WT T cells when stimulated with CD3/CD28 antibodies or allogeneic dendritic cells for upwards of 5 days; however, AMPK-deficient T cells begin to exhibit decreased proliferation past 10 days in culture as well as following adoptive transfer in vivo. AMPK-deficient T cells also decreased expression of the inflammatory cytokine interferon-gamma (IFNγ), while maintaining comparable expression of IL-2, and had limited expression of chemokine receptor CXCR3. Transfer of AMPK-deficient cells to irradiated CD3ε^−/−^ C57BL/6 mice decreased T cell immune reconstitution and in GVHD models, increased survival, and decreased GVHD severity as measured by clinical score.

**Figure 1.**
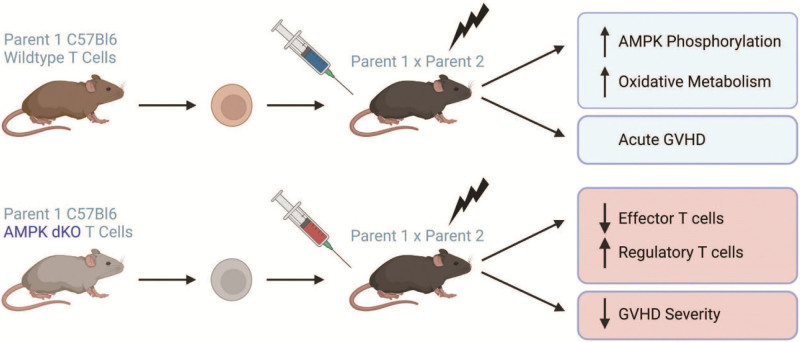
The role of T cell-associated AMPK in promoting GVHD severity. (Top) Wildtype c57Bl/6 T cells transplanted into irradiated (C57Bl/6 x DBA2) F1 recipients increased phosphorylation of AMPK and upregulated oxidative metabolism. This phenotype promoted development of severe acute GVHD. Transplantation of AMPK KO T cells into similar F1 recipients decreased effector T cell numbers and simultaneously increased regulatory T cell percentages (bottom). This favorable regulatory to effector T cell ratio limited GVHD severity with enhanced survival, improved clinical scores, and decreased overall weight loss. Figure created with BioRender.com. AMPK, AMP-activated protein kinase; GVHD, graft-versus-host disease.

Monlish et al ^[11]^ further demonstrated that AMPK activation is highly upregulated in alloreactive T cells during murine GVHD, and that donor T cell deletion of AMPK significantly limited GVHD severity. In concordance with its contemporary study ^[12]^, deletion of AMPKα1/α2 did not impair T cell development and proliferation in vitro, with total thymocyte numbers, splenocyte counts, and CD4^+^/CD8^+^ T cell ratios unaffected by AMPK deficiency. Furthermore, stimulation by both anti-CD3/CD28 antibodies, as well as allogeneic splenocytes in a mixed lymphocyte reaction (MLR), resulted in no differences in proliferation between AMPK-dKO and WT T cells. In both major and minor histocompatibility complex (MHC) models of GVHD, transfer of AMPK-dKO T cells increased survival, decreased weight loss, and lowered GVHD clinical scores out to 60 days post-transplant (Figure 1). Notably, compared to WT cells, fewer AMPK-dKO T cells were recovered in allogeneic recipients on day 7 post-transplant, attributable to both decreased proliferation of CD4 T cells and an increased, early disappearance of CD8 T cells. Importantly, AMPK-dKO and WT T cells were recovered in equal amounts following syngeneic transplantation, a result in line with previous studies ^[43]^. This result underscores the specific necessity for AMPK following allogeneic stimulation, but not necessarily in post-transplant biology as a whole. Interestingly, deletion of AMPK in allogeneic T cells did not hinder FAO, decrease autophagy, or alter mTOR signaling. The effects of AMPK deletion on Treg formation and survival were also investigated, as GVHD severity can be mitigated by increased number and/or adoptive transfer of donor Treg ^[50,51]^. Notably, transplantation of AMPK-dKO T cells increased the number and frequency of post-transplant Treg by 2-fold compared to WT T cells, despite similar Treg frequency and equivalent suppressive function pre-transplant. In addition, co-transplant of WT Treg with AMPK-dKO conventional (non-Treg) cells significantly increased post-transplant WT Treg numbers, suggesting that absence of AMPK in conventional, disease-causing T cells was responsible for the salubrious effects on Treg differentiation and stability.

An ideal therapeutic strategy in alloHSCT would minimize GVHD pathogenesis while preserving GVL effects. In this regard, Monlish et al ^[11]^ demonstrated that AMPK is not necessary for T-cell–dependent anti-leukemic activity, as transplantation of AMPK-dKO T cells in an allogeneic leukemia model reduced tumor burden to a similar degree and promoted equivalent survival of B62DF1 recipients at 4 and 10 weeks post-transplant. In addition, recipient tissues including the liver, spleen, and peripheral blood had equal tumor infiltration in recipients of WT and AMPK-dKO T cells over multiple time points post-transplant and over a 10-fold range of donor T cell doses. Together, these results highlight the potential for AMPK-deficient T cells to ameliorate GVHD while still preserving GVL effects.

## 6. AMPK as a therapeutic target for GVHD

Due to its prominent role in alloreactive T cells, targeting AMPK could represent a promising therapeutic strategy for mitigating GVHD. Thus far, however, AMPK and its contributions to alloreactive T cell biology has largely been studied in murine models. Characterizing the role of AMPK in human T cells during GVHD, as well as the impact of its deficiency on T cell responses and disease pathogenesis, will be necessary to validate the feasibility of targeting AMPK to mitigate GVHD clinically. Monlish et al ^[11]^ conducted preliminary studies demonstrating that AMPK is upregulated in human T cells during both an allogeneic human MLR and also following transplantation of human T cells into a xenogeneic model of GVHD in vivo. It will be important to characterize mitochondrial mass, mitochondrial membrane potential, and metabolic function via extracellular flux analysis in AMPK-deficient human T cells, as these cells would be expected to exhibit a unique mitochondrial phenotype and the potential for altered oxidative metabolism. Furthermore, it is well established that serum levels of IFN-γ increase in patients with acute GVHD ^[52,53]^. As decreased production of pro-inflammatory cytokines in AMPK-deficient cells was shown in previous studies ^[12]^, evaluation of effector function and cytokine profiles in human T cells with and without AMPK is imperative.

Moving forward, ex vivo application of novel gene editing strategies like CRISPR-Cas9 could be employed for the rapid generation of AMPK-deficient T cells for use in both analytical purposes and clinical administration, for example, as an add-back therapy in conjunction with transplantation of CD34^+^ selected grafts ^[54,55]^. Additionally, use of pharmacologic AMPK inhibitors during the cell manufacturing process ^[56]^ may confer similar GVHD-mitigating effects without the potential risks associated with genome engineering ^[57]^. In sum, due to the increased reliance of alloreactive T cells on oxidative metabolism, modulation of AMPK is a promising strategy for minimizing GVHD during alloHSCT, establishing a paradigm that could apply to a host of related auto- and alloimmune disease processes.

## Funding

This work was supported by grants to CAB from the National Institutes of Health (R01HL144556—The Role of AMP-activated Protein Kinase in GVHD-causing T Cells, and K08 HL123631), the American Society of Hematology (ASH)-Scholar award, and the Be the Match Foundation Amy Strelzer Manasevit award. ASR was supported by funding from the ASH Research Training Award for Fellows and the Burroughs Wellcome fund.

## Conflicts of interest

The authors declare that they have no conflicts of interest.

## References

[1] Esiashvili N Lu X Ulin K. Higher reported lung dose received during total body irradiation for allogeneic hematopoietic stem cell transplantation in children with acute lymphoblastic leukemia is associated with inferior survival: a report from the Children’s Oncology Group. Int J Radiat Oncol Biol Phys. 2019;104:513–21. 30807822 10.1016/j.ijrobp.2019.02.034 PMC6548591

[2] Zeiser R Blazar BR. Acute graft-versus-host disease - biologic process, prevention, and therapy. N Engl J Med. 2017;377:2167–79. 29171820 10.1056/NEJMra1609337 PMC6034180

[3] Ferrara JLM Levine JE Reddy P. Graft-versus-host disease. Lancet. 2009;373(9674):1550–61. 19282026 10.1016/S0140-6736(09)60237-3 PMC2735047

[4] MacMillan ML Weisdorf DJ Wagner JE. Response of 443 patients to steroids as primary therapy for acute graft-versus-host disease: comparison of grading systems. Biol Blood Marrow Transplant. 2002;8:387–94. 12171485 10.1053/bbmt.2002.v8.pm12171485

[5] Ullmann AJ Schmidt-Hieber M Bertz H. Infectious diseases in allogeneic haematopoietic stem cell transplantation: prevention and prophylaxis strategy guidelines 2016. Ann Hematol. 2016;95:1435–55. 27339055 10.1007/s00277-016-2711-1 PMC4972852

[6] Li J-M Giver CR Lu Y. Separating graft-versus-leukemia from graft-versus-host disease in allogeneic hematopoietic stem cell transplantation. Immunotherapy. 2009;1:599–621. 20191089 10.2217/imt.09.32 PMC2827928

[7] Rezvani AR Storb RF. Separation of graft-vs.-tumor effects from graft-vs.-host disease in allogeneic hematopoietic cell transplantation. J Autoimmun. 2008;30:172–9. 18242060 10.1016/j.jaut.2007.12.002 PMC2329571

[8] Glick GD Rossignol R Lyssiotis CA. Anaplerotic metabolism of alloreactive T cells provides a metabolic approach to treat graft-versus-host disease. J Pharmacol Exp Ther. 2014;351:298-307. 25125579 10.1124/jpet.114.218099 PMC4201277

[9] Gatza E Wahl DR Opipari AW. Manipulating the bioenergetics of alloreactive T cells causes their selective apoptosis and arrests graft-versus-host disease. Sci Transl Med. 2011;3:67ra–8. 10.1126/scitranslmed.3001975 PMC3364290 21270339

[10] Byersdorfer CA Tkachev V Opipari AW. Effector T cells require fatty acid metabolism during murine graft-versus-host disease. Blood. 2013;122:3230–7. 24046012 10.1182/blood-2013-04-495515 PMC3814737

[11] Monlish DA Beezhold KJ Chiaranunt P. Deletion of AMPK minimizes graft-versus-host disease through an early impact on effector donor T cells. JCI Insight. 2021;6:e143811. 10.1172/jci.insight.143811 PMC8410053 34291733

[12] Lepez A Pirnay T Denanglaire S. Long-term T cell fitness and proliferation is driven by AMPK-dependent regulation of reactive oxygen species. Sci Rep. 2020;10:21673. 33303820 10.1038/s41598-020-78715-2 PMC7728748

[13] Mills EL Kelly B O’Neill LAJ. Mitochondria are the powerhouses of immunity. Nat Immunol. 2017;18:488–98. 28418387 10.1038/ni.3704

[14] Chapman NM Boothby MR Chi H. Metabolic coordination of T cell quiescence and activation. Nat Rev Immunol. 2020;20:55–70. 31406325 10.1038/s41577-019-0203-y

[15] Pearce EL Poffenberger MC Chang C-H. Fueling immunity: insights into metabolism and lymphocyte function. Science. 2013;342(6155):1242454. 24115444 10.1126/science.1242454 PMC4486656

[16] van der Windt GJW O’Sullivan D Everts B. CD8 memory T cells have a bioenergetic advantage that underlies their rapid recall ability. Proc Natl Acad Sci USA. 2013;110:14336–41. 23940348 10.1073/pnas.1221740110 PMC3761631

[17] Corrado M Pearce EL. Targeting memory T cell metabolism to improve immunity. J Clin Invest. 2022;132:e148546. 34981777 10.1172/JCI148546 PMC8718135

[18] Kempkes RWM Joosten I Koenen HJPM. Metabolic pathways involved in regulatory T cell functionality. Front Immunol. 2019;10:2839. 31849995 10.3389/fimmu.2019.02839 PMC6902900

[19] Howie D Cobbold SP Adams E. Foxp3 drives oxidative phosphorylation and protection from lipotoxicity. JCI Insight. 2017;2:e89160. 28194435 10.1172/jci.insight.89160 PMC5291728

[20] Huang Y Si X Shao M. Rewiring mitochondrial metabolism to counteract exhaustion of CAR-T cells. J Hematol Oncol. 2022;15:38. 35346311 10.1186/s13045-022-01255-x PMC8960222

[21] Kawalekar OU O’Connor RS Fraietta JA. Distinct signaling of coreceptors regulates specific metabolism pathways and impacts memory development in CAR T cells. Immunity. 2016;44:380–90. 26885860 10.1016/j.immuni.2016.01.021 PMC13498396

[22] Maude SL Frey N Shaw PA. Chimeric antigen receptor T cells for sustained remissions in leukemia. N Engl J Med. 2014;371:1507–17. 25317870 10.1056/NEJMoa1407222 PMC4267531

[23] Long AH Haso WM Shern JF. 4-1BB costimulation ameliorates T cell exhaustion induced by tonic signaling of chimeric antigen receptors. Nat Med. 2015;21:581–90. 25939063 10.1038/nm.3838 PMC4458184

[24] MacPherson S Keyes S Kilgour MK. Clinically relevant T cell expansion media activate distinct metabolic programs uncoupled from cellular function. Mol Ther Methods Clin Dev. 2022;24:380–93. 35284590 10.1016/j.omtm.2022.02.004 PMC8897702

[25] Kishton RJ Barnes CE Nichols AG. AMPK is essential to balance glycolysis and mitochondrial metabolism to control T-ALL cell stress and survival. Cell Metab. 2016;23:649–62. 27076078 10.1016/j.cmet.2016.03.008 PMC4832577

[26] Weng AP Ferrando AA Lee W. Activating mutations of NOTCH1 in human T cell acute lymphoblastic leukemia. Science. 2004;306(5694):269–71. 15472075 10.1126/science.1102160

[27] Wahl DR Petersen B Warner R. Characterization of the metabolic phenotype of chronically activated lymphocytes. Lupus. 2010;19:1492–501. 20647250 10.1177/0961203310373109

[28] Yin Y Choi S-C Xu Z. Normalization of CD4^+^ T cell metabolism reverses lupus. Sci Transl Med. 2015;7(274):274ra–27418. 10.1126/scitranslmed.aaa0835 PMC5292723 25673763

[29] MacIver NJ Michalek RD Rathmell JC. Metabolic regulation of T lymphocytes. Annu Rev Immunol. 2013;31:259–83. 23298210 10.1146/annurev-immunol-032712-095956 PMC3606674

[30] Levine LS Hiam-Galvez KJ Marquez DM. Single-cell analysis by mass cytometry reveals metabolic states of early-activated CD8^+^ T cells during the primary immune response. Immunity. 2021;54:829–44.e5. 33705706 10.1016/j.immuni.2021.02.018 PMC8046726

[31] Brown RA Byersdorfer CA. Metabolic pathways in alloreactive T cells. Front Immunol. 2020;11:1517. 32793207 10.3389/fimmu.2020.01517 PMC7393946

[32] Hardie DG Ross FA Hawley SA. AMPK: a nutrient and energy sensor that maintains energy homeostasis. Nat Rev Mol Cell Biol. 2012;13:251–62. 22436748 10.1038/nrm3311 PMC5726489

[33] Ma EH Poffenberger MC Wong AH-T. The role of AMPK in T cell metabolism and function. Curr Opin Immunol. 2017;46:45–52. 28460345 10.1016/j.coi.2017.04.004

[34] Mihaylova MM Shaw RJ. The AMPK signalling pathway coordinates cell growth, autophagy and metabolism. Nat Cell Biol. 2011;13:1016–23. 21892142 10.1038/ncb2329 PMC3249400

[35] Xiao B Sanders MJ Underwood E. Structure of mammalian AMPK and its regulation by ADP. Nature. 2011;472(7342):230–3. 21399626 10.1038/nature09932 PMC3078618

[36] Saha AK Ruderman NB. Malonyl-CoA and AMP-activated protein kinase: an expanding partnership. Mol Cell Biochem. 2003;253(1-2):65–70. 14619957 10.1023/a:1026053302036

[37] Inoki K Zhu T Guan K-L. TSC2 mediates cellular energy response to control cell growth and survival. Cell. 2003;115:577–90. 14651849 10.1016/s0092-8674(03)00929-2

[38] Shaw RJ Bardeesy N Manning BD. The LKB1 tumor suppressor negatively regulates mTOR signaling. Cancer Cell. 2004;6:91–9. 15261145 10.1016/j.ccr.2004.06.007

[39] Gwinn DM Shackelford DB Egan DF. AMPK phosphorylation of raptor mediates a metabolic checkpoint. Mol Cell. 2008;30:214–26. 18439900 10.1016/j.molcel.2008.03.003 PMC2674027

[40] Tamás P Hawley SA Clarke RG. Regulation of the energy sensor AMP-activated protein kinase by antigen receptor and Ca^2+^ in T lymphocytes. J Exp Med. 2006;203:1665–70. 16818670 10.1084/jem.20052469 PMC2118355

[41] MacIver NJ Blagih J Saucillo DC. The liver kinase B1 is a central regulator of T cell development, activation, and metabolism. J Immunol. 2011;187:4187–98. 21930968 10.4049/jimmunol.1100367 PMC3206094

[42] Mayer A Denanglaire S Viollet B. AMP-activated protein kinase regulates lymphocyte responses to metabolic stress but is largely dispensable for immune cell development and function. Eur J Immunol. 2008;38:948–56. 18350549 10.1002/eji.200738045

[43] Blagih J Coulombe F Vincent EE. The energy sensor AMPK regulates T cell metabolic adaptation and effector responses in vivo. Immunity. 2015;42:41-–54. 25607458 10.1016/j.immuni.2014.12.030

[44] Rolf J Zarrouk M Finlay DK. AMPKα1: a glucose sensor that controls CD8 T-cell memory. Eur J Immunol. 2013;43:889–96. 23310952 10.1002/eji.201243008 PMC3734624

[45] Pearce EL Walsh MC Cejas PJ. Enhancing CD8 T-cell memory by modulating fatty acid metabolism. Nature. 2009;460(7251):103–7. 19494812 10.1038/nature08097 PMC2803086

[46] Zhu H Liu Z An J. Activation of AMPKα1 is essential for regulatory T cell function and autoimmune liver disease prevention. Cell Mol Immunol. 2021;18:2609–17. 34728795 10.1038/s41423-021-00790-w PMC8632917

[47] Michalek RD Gerriets VA Jacobs SR. Cutting edge: distinct glycolytic and lipid oxidative metabolic programs are essential for effector and regulatory CD4^+^ T cell subsets. J Immunol. 2011;186:3299–303. 21317389 10.4049/jimmunol.1003613 PMC3198034

[48] Fontaine E. Metformin-induced mitochondrial complex I inhibition: facts, uncertainties, and consequences. Front Endocrinol. 2018;9:753. 10.3389/fendo.2018.00753 PMC6304344 30619086

[49] He N Fan W Henriquez B. Metabolic control of regulatory T cell (Treg) survival and function by Lkb1. Proc Natl Acad Sci USA. 2017;114:12542–7. 29109251 10.1073/pnas.1715363114 PMC5703326

[50] Edinger M Hoffmann P Ermann J. CD4^+^CD25^+^ regulatory T cells preserve graft-versus-tumor activity while inhibiting graft-versus-host disease after bone marrow transplantation. Nat Med. 2003;9:1144–50. 12925844 10.1038/nm915

[51] Brunstein CG Miller JS McKenna DH. Umbilical cord blood-derived T regulatory cells to prevent GVHD: kinetics, toxicity profile, and clinical effect. Blood. 2016;127:1044–51. 26563133 10.1182/blood-2015-06-653667 PMC4768428

[52] Zhang C Huang W Zhang P. Dynamic changes in serum cytokine levels and their clinical significance in predicting acute GVHD. Oncotarget. 2017;8:53691–700. 28881843 10.18632/oncotarget.15738 PMC5581142

[53] Nakamura H Komatsu K Ayaki M. Serum levels of soluble IL-2 receptor, IL-12, IL-18, and IFN-gamma in patients with acute graft-versus-host disease after allogeneic bone marrow transplantation. J Allergy Clin Immunol. 2000;106(1 Pt 2):S45–50. 10887333 10.1067/mai.2000.106774

[54] Porta F Comini M Soncini E. CD34^+^ stem cell selection and CD3^+^ T cell add-back from matched unrelated adult donors in children with primary immunodeficiencies and hematological diseases. Transplant Cell Ther. 2021;27:426.e1–9. 33965183 10.1016/j.jtct.2021.01.020

[55] Geyer MB Ricci AM Jacobson JS. T cell depletion utilizing CD34(+) stem cell selection and CD3(+) addback from unrelated adult donors in paediatric allogeneic stem cell transplantation recipients. Br J Haematol. 2012;157:205–19. 22313507 10.1111/j.1365-2141.2012.09048.x

[56] Liu X Chhipa RR Nakano I. The AMPK inhibitor compound C is a potent AMPK-independent antiglioma agent. Mol Cancer Ther. 2014;13:596–605. 24419061 10.1158/1535-7163.MCT-13-0579 PMC3954437

[57] Zhang X-H Tee LY Wang X-G. Off-target effects in CRISPR/Cas9-mediated genome engineering. Mol Ther Nucleic Acids. 2015;4:e264. doi: 10.1038/mtna.2015.37. 26575098 10.1038/mtna.2015.37 PMC4877446

